# Association Between Self-rated Health, Coronary Artery Calcium Scores, and Atherosclerotic Cardiovascular Disease Risk: The Multi-Ethnic Study of Atherosclerosis (MESA)

**DOI:** 10.1001/jamanetworkopen.2018.8023

**Published:** 2019-02-15

**Authors:** Olusola A. Orimoloye, Mohammadhassan Mirbolouk, S. M. Iftekhar Uddin, Zeina A. Dardari, Michael D. Miedema, Mouaz H. Al-Mallah, Joseph Yeboah, Ron Blankstein, Khurram Nasir, Michael J. Blaha

**Affiliations:** 1 Johns Hopkins Ciccarone Center for the Prevention of Heart Disease, Johns Hopkins School of Medicine, Baltimore, Maryland; 2 Minneapolis Heart Institute, Abbott Northwestern Hospital, Minneapolis, Minnesota; 3 King Saud bin Abdulaziz University for Health Sciences, King Abdullah International Medical Research Center, King Abdulaziz Cardiac Center, Ministry of National Guard, Health Affairs, Riyadh, Saudi Arabia; 4 Department of Cardiology, Wake Forest School of Medicine, Winston-Salem, North Carolina; 5 Department of Medicine, Brigham and Women’s Hospital, Boston, Massachusetts; 6 Center for Outcomes Research and Evaluation, Yale School of Medicine, New Haven, Connecticut

## Abstract

**Importance:**

The interplay of self-rated health (SRH), coronary artery calcium (CAC) scores, and cardiovascular risk is poorly described.

**Objectives:**

To assess the degree of correlation between SRH and CAC, to determine whether these measures are complementary for risk prediction, and to assess the incremental value of the addition of SRH to established risk tools.

**Design, Setting, and Participants:**

The Multi-Ethnic Study of Atherosclerosis (MESA) is a large population-based prospective cohort study of adults aged 45 to 84 years who were recruited from 6 US communities. A total of 6764 participants without baseline cardiovascular disease (CVD) were included in the analysis. Data were collected from July 2000 through August 2002. Follow-up was completed by December 2013, and data were analyzed from October 2018 to December 2018.

**Exposures:**

The EVGGFP (excellent, very good, good, fair, and poor) self-assessment of overall health (assessed before the baseline study examination) and CAC score. The EVGGFP rating was categorized as poor/fair, good, very good, or excellent.

**Main Outcomes and Measures:**

Hard coronary heart disease (CHD) events, hard CVD events, and all-cause mortality during a median follow-up of 13.2 years (interquartile range, 12.7-13.7 years).

**Results:**

Among the study population of 6764 participants, the mean (SD) age was 62.1 (10.2) years, and 52.9% were women. The EVGGFP rating was strongly associated with age, sex, race/ethnicity, educational and income levels, healthy diet and physical activity, and cardiovascular risk factors. Despite encapsulating many risk variables, no correlation (*r* = −0.007; *P* = .57) or association between EVGGFP and the presence (χ^2^ = 0.84; *P* = .84) or severity (χ^2^ = 4.64; *P* = .86) of CAC was found. During follow-up, 1161 deaths, 637 hard CVD events, and 405 hard CHD events were recorded. In models adjusted for age, sex, race/ethnicity, and CAC, participants who reported excellent health had a 45% lower risk of CVD (hazard ratio [HR], 0.55; 95% CI, 0.39-0.77) and a 42% lower risk of CHD (HR, 0.58; 95% CI, 0.37-0.90) compared with those who reported poor/fair health. Participants in the excellent SRH category who had any CAC had markedly elevated risk of hard CHD (HR, 6.19; 95% CI, 2.1-18.3) and CVD (HR, 6.50; 95% CI, 2.7-15.6) events compared with those with a CAC score of 0. The addition of the EVGGFP rating to CAC improved the area under the curve (C statistic) for CHD events (0.725 vs 0.734; *P* = .007), CVD events (0.693 vs 0.706; *P* < .001), and all-cause mortality (0.685 vs 0.707; *P* < .001). However, the addition of the EVGGFP rating to the combination of CAC and atherosclerotic CVD risk score did not significantly improve C statistics for CHD events (0.751 vs 0.753; *P* = .39), CVD events (0.739 vs 0.741; *P* = .18), or all-cause mortality (0.779 vs 0.781; *P* = .13).

**Conclusions and Relevance:**

Although SRH and CAC integrate many risk variables, this study suggests that they are poorly correlated and have complementary predictive utility. A perception of excellent health does not obviate the need for definitive assessment of CVD risk, whereas fair/poor perceived health may serve as a risk enhancer, arguing for advanced risk assessment in selected clinical scenarios.

## Key Points

**Question:**

Is self-rated health correlated with the presence of coronary artery calcium, and is it complementary for risk prediction?

**Findings:**

In this cohort study of a large, multiethnic sample of 6764 US adults, self-rated health and coronary artery calcium score were each excellent risk integrators but were poorly correlated with each other. Self-rated health and coronary artery calcium score showed complementary predictive utility, and their simple combination showed similar risk discrimination for coronary heart disease and cardiovascular disease events compared with the 2013 College of Cardiology/American Heart Association atherosclerotic cardiovascular disease risk score.

**Meaning:**

The findings suggest that self-rated health and coronary artery calcium capture largely distinct domains of comprehensive cardiovascular health and may be predictive of risk independent of each other, and self-rated health may have value as a risk enhancer in patients with borderline to intermediate risk.

## Teaser

This population-based cohort study of US adults from the Multi-Ethnic Study of Atherosclerosis (MESA) assesses the degree of correlation between self-rated health and coronary artery calcium and whether these measures are complementary for their association with risk of atherosclerotic cardiovascular disease (CVD).

## Introduction

Self-rated health (SRH) is a popular measure of individuals’ evaluation of their health status.^1,2^ Self-rated health has been described as a cognitive summary of the effects of current and past ailments on health or a comparative rate of decline of bodily function to that of one’s peers, within the context of personality, age, and cultural influences.^3^ Previous studies have shown SRH to be a reproducible measure^4^ that is associated with cardiovascular risk factors and with cardiovascular and all-cause mortality.^3,5,6,7,8^

The coronary artery calcium (CAC) score, a cardiac computed tomographic–derived assessment of the burden of calcified coronary atherosclerosis, is an integrative measure that captures the downstream effects of measured and unmeasurable cardiovascular risk factors.^9^ However, despite a general association with risk factors, CAC burden is heterogeneous across risk factor groups,^10^ and individuals may be poor at estimating their burden of CAC.

Several studies have assessed the utility of each of these measures for mortality and cardiovascular disease (CVD) risk prediction.^7,11^ However, the association between SRH and the true burden of atherosclerosis, as estimated by CAC, and the interplay of these measures for risk estimation have not been explored. In addition, significant interest in formally assessing the incremental benefit of SRH in clinical cardiovascular risk prediction modeling has been indicated.^12^ We therefore sought to assess the association between the standardized EVGGFP (excellent, very good, good, fair, and poor) measure of SRH^13^ and CAC, the complementary value of each for risk prediction, and the incremental value of the addition of SRH to clinical CVD risk prediction.

## Methods

### Study Population

We used data from the Multi-Ethnic Study of Atherosclerosis (MESA), a large, population-based diverse prospective cohort study of 6814 men and women aged 45 to 84 years without CVD at baseline. Participants were recruited from 6 US communities (Baltimore, Maryland; Chicago, Illinois; Forsyth County, North Carolina; Los Angeles County, California; Northern Manhattan, New York; and St Paul, Minnesota) from July 2000 through August 2002. Institutional review boards at all participating centers approved the study, and all participants gave written informed consent.^14^ This study followed the Strengthening the Reporting of Observational Studies in Epidemiology (STROBE) reporting guidelines.

Other details of the rationale and design of the MESA study have been previously described.^14^ After excluding participants without information on SRH (n = 50), we included a total of 6764 MESA participants in our analytic sample.

### Measurement of SRH

An EVGGFP measure of SRH was assessed in MESA before CAC scoring and other baseline examinations via the single question: “Would you say, in general, your health is poor, fair, good, very good, or excellent?” For the purpose of our analysis, participant SRH was grouped into poor/fair, good, very good, and excellent categories.

### CAC Score Measurement

For CAC scoring, each participant underwent 2 consecutive noncontrast cardiac-gated computed tomographic scans at baseline (examination 1). The burden of calcified coronary atherosclerosis in each scan was quantified by 2 independent readers using the Agatston method, with mean results calculated.^15^ Three sites used an electron beam computed tomographic scanner (Imatron C–150XL; GE-Imatron), whereas 3 sites used a 4-slice multidetector computed tomographic scanner. To assess the association between CAC and SRH and for risk prediction, CAC was handled in categorical (0, 1-99, 100-399, and ≥400) and log-transformed continuous (ln [CAC score + 1]) forms, respectively.

### Other Measurements

Baseline information on sociodemographic variables such as age, sex, race/ethnicity, educational status, and income was collected by trained interviewers using standardized questionnaires. Smoking status, alcohol consumption, family history of CVD, and the presence of medical conditions were also assessed using standardized questionnaires. Current smoking was defined as having smoked a cigarette in the past 30 days. Medication use was based on medication inventory.

Physical activity was measured using a detailed semiquantitative questionnaire, and diet was captured using a food frequency questionnaire. Depression was measured using the Center for Epidemiologic Studies Depression Scale. Anthropometric measures were recorded using standard techniques. Body mass index was calculated as weight in kilograms divided by height in meters squared.

In the MESA study, resting blood pressure was measured 3 times in the seated position using an automated oscillometric sphygmomanometer, with the mean of the last 2 readings recorded.^14^ We defined participants with systolic blood pressure of at least 140 mm Hg, with diastolic blood pressure of at least 90 mm Hg, or who were using antihypertensives as having hypertension. Diabetes was defined as self-reported history of diabetes, use of diabetes medications, or a fasting glucose level of at least 126 mg/dL (to convert to millimoles per liter, multiply by 0.0555). Total and high-density lipoprotein cholesterol levels were measured from blood samples taken after a 12-hour fast. Low-density lipoprotein cholesterol level was estimated according to the Friedewald equation.^14^ Atherosclerotic CVD (ASCVD) risk scores for each participant were calculated according to the 2013 American College of Cardiology/American Heart Association (ACC/AHA) pooled cohort equations (PCE).^16^

### Event Ascertainment

Coronary heart disease (CHD) and CVD events were independently adjudicated by 2 physician reviewers and recorded during a median follow-up of 13.2 years (interquartile range, 12.7-13.7 years). Hard CHD events included myocardial infarction, resuscitated cardiac arrest, or CHD death, whereas hard CVD events additionally included stroke and stroke death.^14^ Full details of the MESA methods and adjudication procedures are available on the MESA website.^17^

### Statistical Analysis

Data were analyzed from October 2018 to December 2018. Baseline sociodemographic characteristics, risk factors, and risk measure distributions were compared across SRH categories using χ^2^ and 1-way analysis of variance tests for categorical and continuous variables, respectively. The degree of correlation between EVGGFP rating and CAC was assessed using the Spearman rank correlation test.

To assess the predictive value of SRH (as measured via the EVGGFP rating), Cox proportional hazards regression models were used to estimate hazard ratios (HRs) for each outcome of interest, first in unadjusted models and then in sequentially adjusted models. Model 1 was adjusted for age, sex, and race/ethnicity. Model 2 (adjusted for variables in model 1 and CAC group) was then constructed to assess whether the predictive value of SRH was independent of CAC. To assess whether the predictive value of SRH was retained after adjustment for CAC and cardiovascular risk factors, model 2 was additionally adjusted for hypertension, diabetes, use of medications to lower lipid levels, current cigarette smoking, and family history of CVD (model 3).

To assess the improvement in discrimination on addition of SRH to standard clinical risk prediction tools, we conducted several analyses. First, we reported standard area under the curve (C statistic) for SRH, CAC, and ASCVD risk scores alone. We then assessed relative improvement in risk discrimination on addition of SRH separately to CAC and the ASCVD risk score. This analysis was accomplished by comparing C statistics in models with and without SRH added to CAC (ie, CAC vs CAC plus SRH) and ASCVD risk score (ASCVD risk score vs ASCVD risk score plus SRH).

In addition, we compared C statistics of the combination of CAC plus ASCVD risk score, with and without addition of SRH (ie, CAC plus ASCVD risk score vs CAC plus ASCVD risk score plus SRH). Finally, we directly compared the discriminatory performance of the combination of SRH plus CAC with that of the ASCVD risk score alone (ie, CAC plus SRH vs ASCVD risk score).

We also conducted net reclassification improvement (NRI) analyses, cross-tabulating the PCE with and without the SRH.^18^ The NRI analyses were conducted by using recalibrated 5.0%, 7.5%, and 20.0% ASCVD risk cutoffs^19^ consistent with the new 2018 ACC/AHA risk prediction guidelines.^20^

In further analyses, we assessed the predictive utility of CAC for each outcome of interest within the excellent SRH category using Cox proportional hazards regression models. Models were adjusted for age, sex, race/ethnicity, hypertension, diabetes, use of medications to lower lipid levels, cigarette smoking, and family history of CVD. Similar analyses were conducted for other SRH categories. An interaction term was additionally specified to assess whether the association between CAC and outcomes varied by SRH category. Statistical analysis was performed using Stata software (version 14.0; StataCorp LP); a 2-tailed *P* value <.05 was considered statistically significant.

## Results

In the study population of 6764 participants, the mean (SD) age was 62.1 (10.2) years; 3581 were women (52.9%) and 3183 were men (47.1%). Participants who reported very good and excellent health were younger (mean [SD] age, 61.1 [10.3] and 61.1 [9.8] years, respectively), more likely to be men (1101 [48.6%] and 562 [52.4%], respectively), and more likely to have at least a high school education (2028 [89.5%] and 1013 [94.4%], respectively). Black (261 [41.2%]) and Hispanic (258 [40.8%]) participants were more likely to self-report poor/fair health. There were notable associations between SRH and diet, exercise, and traditional cardiovascular risk factors, which were graded across EVGGFP categories (Table 1). However, there was no association between EVGGFP rating and the presence (χ^2^ = 0.84; *P* = .84) or degree (χ^2^ = 4.64; *P* = .86) of CAC. In addition, there was no significant correlation between EVGGFP rating and CAC groups (*r* = −0.007; *P* = .57).

**Table 1.** Baseline Characteristics of Study Population by SRH Categories

Characteristic	SRH Category				*P* Value
	Poor/Fair (n = 633)	Good (n = 2792)	Very Good (n = 2266)	Excellent (n = 1073)	
Sociodemographic					
Age, mean (SD), y	63.9 (10.1)	63.0 (10.3)	61.1 (10.3)	61.1 (9.8)	<.001
Female, No. (%)	391 (61.8)	1544 (55.3)	1165 (51.4)	481 (44.8)	<.001
Race/ethnicity, No. (%)					
White	95 (15.0)	661 (23.7)	1152 (50.8)	701 (65.3)	<.001
Chinese	19 (3.0)	528 (18.9)	218 (9.6)	31 (2.9)	
Black	261 (41.2)	839 (30.1)	566 (25.0)	211 (19.7)	
Hispanic	258 (40.8)	764 (27.4)	330 (14.6)	130 (12.1)	
High school educational level, No. (%)^a^	373 (58.9)	2116 (75.8)	2028 (89.5)	1013 (94.4)	<.001
Income >$40 000/y, No. (%)	91 (14.4)	764 (27.4)	1079 (47.6)	641 (59.7)	<.001
Risk factors					
BMI, mean (SD), mm Hg	30.1 (6.1)	28.5 (5.7)	28.2 (5.3)	27.1 (4.5)	<.001
Blood pressure, mean (SD)					
Systolic	131.6 (22.8)	129.0 (22.3)	124.7 (20.5)	121.3 (19.0)	<.001
Diastolic	72.8 (10.6)	72.1 (10.4)	71.8 (10.1)	71.1 (10.0)	<.001
Hypertension, No. (%)^b^	427 (67.4)	1507 (54.0)	1003 (44.3)	338 (31.5)	<.001
HDL-C level, mean (SD), mg/dL^c^	50.4 (15.1)	49.8 (13.9)	51.6 (15.1)	53.0 (16.1)	<.001
LDL-C level, mean (SD), mg/dL^c^	115 (32.6)	116.4 (32.3)	118.4 (31.0)	117.7 (29.5)	.03
Total cholesterol level, mean (SD), mg/dL^c^	193.2 (39.2)	193.2 (36.5)	195.6 (34.8)	193.9 (33.3)	.01
Diabetes, No. (%)^d^	171 (27.0)	454 (16.3)	180 (7.9)	48 (4.5)	<.001
Family history of CHD, No. (%)^e^	263 (41.5)	1077 (38.6)	935 (41.3)	435 (40.5)	.36
Smoking status, No. (%)					
Never	308 (49.0)	1475 (53.0)	1113 (49.3)	493 (46.0)	<.001
Former	202 (32.1)	941 (33.8)	858 (38.0)	473 (44.2)	
Current	119 (18.9)	367 (13.2)	288 (12.7)	105 (9.8)	
Current alcohol use, No. (%)^f^	248 (39.2)	1329 (47.6)	1371 (60.5)	773 (72.0)	<.001
Healthy diet, No. (%)^g^	209 (33.1)	1144 (41.0)	1095 (48.3)	604 (56.3)	<.001
Healthy physical activity, No. (%)^h^^,^^i^	67 (10.6)	344 (12.3)	459 (20.3)	326 (30.4)	<.001
Depression, No. (%)^j^	228 (36.0)	568 (20.3)	387 (17.1)	144 (13.4)	<.001
Medication use, No. (%)					
Antihypertensives	369 (58.3)	1167 (41.8)	766 (33.8)	212 (19.8)	<.001
Lipid level lowering	121 (19.1)	475 (17.0)	364 (16.1)	133 (12.4)	.001
β-Blockers	87 (13.7)	281 (10.1)	192 (8.5)	44 (4.1)	<.001
Risk measures					
CAC presence, No. (%)	310 (49.0)	1408 (50.4)	1119 (49.4)	530 (49.4)	.84
CAC score, No. (%)					
0	323 (51.0)	1384 (49.6)	1147 (50.6)	543 (50.6)	.86
1-99	163 (25.8)	737 (26.4)	598 (26.4)	278 (25.9)	
100-399	75 (11.8)	394 (14.1)	299 (13.2)	151 (14.1)	
≥400	72 (11.4)	277 (9.9)	222 (9.8)	101 (9.4)	
CAC score, mean (SD)	155.1 (449.1)	140.6 (389.1)	152.4 (454.5)	138.8 (369.7)	.96
ASCVD risk score, mean (SD), %^k^	18.2 (15.5)	15.1 (14.1)	12.0 (12.1)	10.6 (10.7)	<.001
ASCVD category, No. (%)					
<7.5%	196 (31.4)	1070 (38.6)	1075 (47.8)	565 (53.2)	<.001
7.5%-15%	145 (23.2)	615 (22.2)	537 (23.9)	226 (21.3)	
>15%	284 (45.4)	1087 (39.2)	635 (28.3)	272 (25.6)	

Abbreviations: ASCVD, atherosclerotic cardiovascular disease; BMI, body mass index (calculated as weight in kilograms divided by height in meters squared); CAC, coronary artery calcium; CHD, coronary heart disease; HDL-C, high-density lipoprotein cholesterol; LDL-C, low-density lipoprotein cholesterol; SRH, self-rated health.

^a^ Data were missing for 23 individuals.

^b^ Defined as systolic blood pressure of at least 140 mm Hg, diastolic blood pressure of at least 90 mm Hg, or use of antihypertensives.

^c^ To convert cholesterol levels to millimoles per liter, multiply by 0.0259.

^d^ Data were missing for 24 individuals.

^e^ Data were missing for 418 individuals.

^f^ Data were missing for 51 individuals.

^g^ Data were missing for 282 individuals.

^h^ Defined as greater than 150 min/wk of moderate intensity or 75 min/wk of vigorous intensity activity.

^i^ Data were missing for 19 individuals.

^j^ Defined as Center for Epidemiologic Studies Depression Scale score of at least 16 or use of antidepressants.

^k^ Derived according to the 2013 American College of Cardiology/American Heart Association pooled cohort equations.

During a median follow-up of 13.2 years, 1161 deaths, 637 hard CVD events, and 405 hard CHD events were recorded. Better SRH was associated with a graded decrease in risk across all outcomes, which persisted after sequential adjustment (Table 2). In age-, sex-, race-, and CAC-adjusted models, participants who self-reported excellent health had at least a 45% lower risk of CVD (HR, 0.55; 95% CI, 0.39-0.77) and 42% lower risk of CHD (HR, 0.58; 95% CI, 0.37-0.90) compared with those who self-reported poor/fair health. Risk estimates were attenuated in models accounting for traditional risk factors (Table 2).

**Table 2.** Associations Between SRH and Incident Events

SRH	Outcome, HR (95% CI)		
	All-Cause Death	Hard CVD Event	Hard CHD Event
**Unadjusted Models**			
Poor/fair	1 [Reference]	1 [Reference]	1 [Reference]
Good	0.65 (0.54-0.77)	0.77 (0.60-0.99)	0.89 (0.64-1.23)
Very good	0.54 (0.45-0.65)	0.63 (0.49-0.82)	0.69 (0.49-0.97)
Excellent	0.36 (0.28-0.45)	0.47 (0.34-0.64)	0.53 (0.35-0.80)
**Model 1**^**a**^			
Poor/fair	1 [Reference]	1 [Reference]	1 [Reference]
Good	0.66 (0.55-0.79)	0.84 (0.65-1.08)	0.93 (0.67-1.30)
Very good	0.57 (0.47-0.70)	0.73 (0.55-0.96)	0.75 (0.52-1.07)
Excellent	0.36 (0.29-0.47)	0.51 (0.36-0.72)	0.54 (0.35-0.83)
**Model 2**^**b**^			
Poor/fair	1 [Reference]	1 [Reference]	1 [Reference]
Good	0.62 (0.52-0.74)	0.79 (0.61-1.02)	0.89 (0.64-1.23)
Very good	0.58 (0.48-0.70)	0.74 (0.56-0.97)	0.76 (0.53-1.09)
Excellent	0.37 (0.29-0.47)	0.55 (0.39-0.77)	0.58 (0.37-0.90)
**Model 3**^**c**^			
Poor/fair	1 [Reference]	1 [Reference]	1 [Reference]
Good	0.71 (0.58-0.85)	0.92 (0.70-1.20)	1.06 (0.74-1.51)
Very good	0.66 (0.54-0.82)	0.91 (0.68-1.21)	0.93 (0.63-1.37)
Excellent	0.45 (0.34-0.58)	0.71 (0.50-1.02)	0.78 (0.49-1.24)

Abbreviations: CHD, coronary heart disease; CVD, cardiovascular disease; HR, hazard ratio; SRH, self-rated health.

^a^ Adjusted for age, sex, and race/ethnicity.

^b^ Adjusted for coronary artery calcium group, age, sex, and race/ethnicity.

^c^ Adjusted for coronary artery calcium group, age, sex, and race/ethnicity, hypertension (defined as systolic blood pressure ≥140 mm Hg, diastolic blood pressure ≥90 mm Hg, or use of antihypertensives), diabetes, use of medications to lower lipid levels, cigarette smoking, and family history of CVD.

Increasing CAC was associated with higher risk in all SRH groups, including excellent SRH (Table 3 and eTables 1-4 in the Supplement). In models adjusted for age, sex, race/ethnicity, hypertension, diabetes, use of medications to lower lipid levels, cigarette smoking, and family history of CVD, participants in the excellent EVGGFP category who had any CAC had a markedly elevated risk of hard CHD (HR, 6.19; 95% CI, 2.10-18.30) and CVD (HR, 6.50; 95% CI, 2.70-15.60) events compared with those with a CAC score of 0 (Table 3). There was no significant interaction between CAC and SRH for the prediction of incident CHD events, CVD events, or all-cause death.

**Table 3.** Association Between CAC and Incident Events Among Individuals With Excellent Self-rated Health

Characteristic	HR (95% CI)		
	All-Cause Death	Hard CVD Events	Hard CHD Events
**Adjusted for Age, Sex, and Race/Ethnicity**			
CAC score			
0	1 [Reference]	1 [Reference]	1 [Reference]
1-99	1.75 (1.02-3.00)	8.56 (3.50-20.89)	7.59 (2.51-22.98)
100-399	2.13 (1.19-3.80)	8.41 (3.15-22.44)	8.55 (2.54-28.76)
≥400	1.76 (0.93-3.32)	10.90 (3.96-29.97)	15.0 (4.41-51.03)
Presence of CAC (yes vs no)	1.86 (1.13-3.04)	8.76 (3.67-20.97)	8.51 (2.92-24.79)
Log-transformed (CAC score + 1)	1.10 (1.02-1.20)	1.34 (1.20-1.49)	1.41 (1.22-1.63)
**Adjusted for Risk Factors**^**a**^			
CAC score			
0	1 [Reference]	1 [Reference]	1 [Reference]
1-99	1.66 (0.94-2.93)	6.46 (2.60-16.06)	5.72 (1.84-17.77)
100-399	2.03 (1.11-3.73)	5.81 (2.12-15.89)	5.75 (1.67-19.76)
≥400	1.93 (0.98-3.80)	7.76 (2.76-21.83)	9.40 (2.70-32.77)
Presence of CAC (yes vs no)	1.81 (1.08-3.03)	6.45 (2.66-15.63)	6.19 (2.09-18.31)
Log-transformed (CAC score + 1)	1.11 (1.02-1.21)	1.28 (1.13-1.44)	1.34 (1.15-1.56)

Abbreviations: CAC, coronary artery calcium; CHD, coronary heart disease; CVD, cardiovascular disease; HR, hazard ratio.

^a^ Includes age, sex, and race/ethnicity, hypertension (defined as systolic blood pressure ≥140 mm Hg, diastolic blood pressure ≥90 mm Hg, or use of antihypertensives), diabetes, use of medications to lower lipid levels, cigarette smoking, and family history of CVD.

In models adjusted for age, race/ethnicity, and sex, a graded reduction in the risk of incident events with better SRH was noted among individuals with CAC of 0. Statistical significance was noted for CHD (HR, 0.21; 95% CI, 0.06-0.70) and CVD (HR, 0.20; 95% CI, 0.08-0.52) events in the excellent SRH group only (eTable 5 in the Supplement).

For all outcomes of interest, the ASCVD risk score and CAC had higher C statistics than the EVGGFP measure in univariate analysis (eTable 6 in the Supplement). The addition of SRH to CAC significantly improved C statistics for CHD events (0.725 vs 0.734; *P* = .007), CVD events (0.693 vs 0.706; *P* < .001), and all-cause mortality (0.685 vs 0.707; *P* < .001). In contrast, the addition of EVGGFP rating to the ASCVD risk score did not significantly improve C statistics for CHD events (0.712 vs 0.711; *P* = .83), CVD events (0.718 vs 0.717; *P* = .74), or all-cause mortality (0.777 vs 0.777; *P* = .80) (Table 4). Similarly, NRI analysis for the addition of EVGGFP rating to PCE alone showed no significant improvement in CHD or CVD risk classification across recalibrated 5.0%, 7.5%, and 20.0% risk thresholds. The addition of EVGGFP rating to CAC score, however, resulted in slight improvement in classification for CHD events (NRI = 0.04; *P* = .05) and CVD events (NRI = 0.03; *P* = .02).

**Table 4.** Comparison of C Statistics for Clinical Risk Tools With and Without SRH, for the Prediction of Hard CHD Events, Hard CVD Events, and All-Cause Death

Risk Tools	Hard CHD Events			Hard CVD Events			All-Cause Death		
	Risk Tool	Risk Tool Plus SRH^a^	*P* Value	Risk Tool	Risk Tool Plus SRH^a^	*P* Value	Risk Tool	Risk Tool Plus SRH^a^	*P* Value
PCE	0.712	0.711	.83	0.718	0.717	.74	0.777	0.777	.80
CAC	0.725	0.734	.007	0.693	0.706	<.001	0.685	0.707	<.001

Abbreviations: CAC, coronary artery calcium; CHD, coronary heart disease; CVD, cardiovascular disease; PCE, 2013 American College of Cardiology/American Heart Association pooled cohort equations; SRH, self-rated health.

^a^ Measured using the EVGGFP (excellent, very good, good, fair, and poor) rating of overall health.

A comparison of risk discrimination between the combination of CAC score, SRH vs the PCE showed similar risk discrimination for CHD (C statistic, 0.734 vs 0.712; *P* = .09) and CVD (C statistic, 0.706 vs 0.717; *P* = .31) events (eTable 7 in the Supplement). Addition of the EVGGFP measure to the combination of the PCE and CAC score, however, showed no significant improvement in C statistics across all outcomes, including all-cause death (eTable 8 in the Supplement).

## Discussion

In an ethnically diverse, community-based cohort, we showed a strong association of SRH with physical activity, healthy diet, and CVD risk factors, but no association or correlation between SRH and CAC. Although we found SRH to be predictive of CVD events and all-cause mortality, we showed that CAC score still risk stratifies in all SRH categories, including excellent health. In addition, we showed that although the addition of SRH to CAC score improves CHD and CVD risk discrimination compared with CAC score alone, the routine addition of SRH to the clinically recommended combination of the PCE and CAC score did not significantly improve risk discrimination for CHD or CVD events. However, we demonstrated that a simple combination of the EVGGFP measure of SRH and CAC score has similar risk discrimination for CVD and CHD outcomes when compared with the PCE alone.

Several key findings from our study merit discussion. First, similar to prior studies, our analysis showed that SRH is associated with incident CVD events and all-cause mortality.^6,7,8^ However, our study extends prior knowledge by examining, for the first time, the predictive value of SRH in the context of existing risk tools such as the PCE and CAC score, seeking to assess combinations of SRH, PCE, and CAC score that may improve current clinical risk assessment or provide quick and less expensive strategies for risk prediction in selective screening programs, population health management, research, or other settings where decisions are made based on risk. Importantly, SRH can easily be assessed electronically inside or outside the health care setting; therefore, its applicability is broad.

Next, we showed that even among people who self-reported excellent health, 101 (9.4%) had markedly elevated CAC score. Because individuals’ perception of their health may influence their presentation to health care professionals (those who perceive themselves to be in excellent health may be less likely to interact with the health care system),^21^ we believe that our unique finding of the absence of correlation or association between CAC score and SRH may serve as an important public health message. For example, this message may further motivate individuals to consider advanced and definitive risk assessment, even when they deem themselves to be in excellent health.

Our results should be approached from the perspective of risk integration, as illustrated in the Figure. The 2018 ACC/AHA guidelines recommend a global risk assessment approach to CVD risk prediction, based on the PCE, a probability-based product of the integration of known traditional risk factors.^16^ Similarly, the predictive utility of CAC score ensues from its ability to capture the downstream effects of measurable and unmeasured CVD risk factors, as well as the effect of biological variables such as genetic predispositions to CVD.^22^ However, the occurrence of clinical CVD is multifactorial, and multiple psychological and social factors that are not captured by these risk tools may contribute to the occurrence or progression of CVD. For example, several prospective studies have shown associations between social determinants of health, including depression, social isolation, and anxiety, and CVD,^23^ and these factors are not captured by current risk tools. In the context of this gap in risk prediction, we believe that SRH, given its ability to integrate these variables (in addition to classic risk variables), may be a useful complement in CVD risk prediction.

**Figure.**
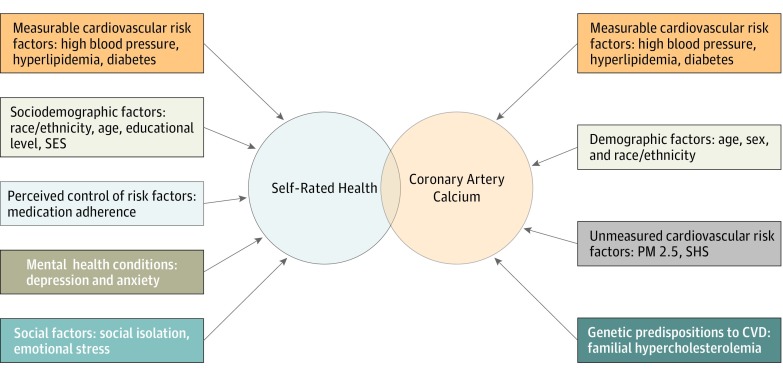
Theoretical Framework of Risk Domains, Self-rated Health, and Coronary Artery Calcium Score PM 2.5 indicates fine particulate matter with diameter of 2.5 μm or smaller; SES, socioeconomic status; and SHS, secondhand smoke.

Therefore, we posit that the residual predictive ability of SRH after adjustment for CAC and its attenuation after adjustment for cardiovascular risk factors lends credence to the hypothesis that although some overlap exists between the risk domains captured by the CAC score and SRH (eg, traditional cardiovascular risk factors), there are distinct differences in the scope of risk integration by these measures. For example, although CAC score is known to capture unmeasured risk factors such as genetic predisposition, it does not capture social or psychological elements such as depression and social isolation, which also are associated with CVD risk.^24^ The strong association of SRH with traditional CVD risk factors and the attenuation of its predictive value for CVD and CHD outcomes in models accounting for risk factors highlight the potential utility of SRH as a cardiovascular risk integrator whose predictive value is partly explained by individuals’ cognitive summarization of the health effects of CVD risk factors known to them.^25^ We believe that the improvement in C statistics noted across all outcomes on adding SRH to CAC score, the predictive value of CAC score in the excellent SRH group, and the residual predictive value of SRH in the group with a CAC score of 0 further hint at the complementarity of these measures for risk prediction.

### Clinical Implications

Owing to multiple studies confirming the association between SRH and incident CVD outcomes, calls in the literature have been made to investigate its possible role in risk stratification, in addition to clinical risk tools.^12^ To our knowledge, this study is the first to investigate the incremental value of SRH in addition to CAC score and the PCE. We found that the addition of SRH to the combination of CAC score and the PCE did not significantly improve risk discrimination, arguing for more selective consideration of SRH in clinical practice. Under the rubric of the new 2018 ACC/AHA risk prediction guidelines, we propose that poor SRH may be useful as a risk enhancer in patients at borderline to intermediate risk (5%-20% ASCVD), which may suggest a potential need for more definitive risk assessment using tools such as CAC scoring. Importantly, however, excellent SRH may not be a reliable marker of very low risk in a patient.

### Strengths and Limitations

To our knowledge, this study is the first to assess the interplay of SRH—which can easily be assessed in clinical practice—and CAC for ASCVD risk prediction. A notable limitation is that the predictive utility of SRH may depend on context, because it is mostly a subjective measure that may depend on the setting in which information is collected or the specific question that is asked. Furthermore, participants’ responses may be influenced by their educational status or prior interactions with the clinical environment.^7^ Validation studies are therefore needed using similar simple measures of SRH across different populations.

## Conclusions

Self-rated health and CAC score can be viewed as useful clinical risk integrators, yet this study suggests that they are poorly correlated and appear to be complementary. The combination of SRH and CAC score may provide similar risk discrimination to the PCE and may be a useful and parsimonious approach to initial risk prediction in screening programs, in research studies, and, selectively, in clinical practice.

## Notes

Supplement. **eTable 1.** Cumulative Incidence of Events by CAC Score Categories, Among Individuals Who Reported Excellent Self-rated Health **eTable 2.** Association Between CAC and Incident Events Among Individuals Who Reported Very Good Self-rated Health **eTable 3.** Association Between CAC and Incident Events Among Individuals Who Self-Reported Good Self-rated Health **eTable 4.** Association Between CAC and Incident Events Among Individuals Who Self-Reported Poor/Fair Health **eTable 5.** Association Between Self-rated Health and Incident Events Among Individuals With CAC Score of 0 **eTable 6.** AUC C Statistics for Established Clinical Risk Tools and Self-rated Health for the Prediction of Hard CHD Events, Hard CVD Events, and All-Cause Death **eTable 7.** Comparison of AUC C Statistics for the Combination of CAC and EVGGFP vs PCE **eTable 8.** Comparison of AUC C Statistics for Combination of PCE and CAC With and Without Self-rated Health
