# Supplementary material for: Association Between Self-rated Health, Coronary Artery Calcium Scores, and Atherosclerotic Cardiovascular Disease Risk: The Multi-Ethnic Study of Atherosclerosis (MESA)
Source: JAMA Netw Open. 2019 Feb 15;2(2):e188023. doi: 10.1001/jamanetworkopen.2018.8023 (PMC6484585; doi:10.1001/jamanetworkopen.2018.8023)
Supplement: Supplement. — eTable 1. Cumulative Incidence of Events by CAC Score Categories, Among Individuals Who Reported Excellent Self-rated Health eTable 2. Association Between CAC and Incident Events Among Individuals Who Reported Very Good Self-rated Health eTable 3. Association Between CAC and Incident Events Among Individuals Who Self-Reported Good Self-rated Health eTable 4. Association Between CAC and Incident Events Among Individuals Who Self-Reported Poor/Fair Health eTable 5. Association Between Self-rated Health and Incident Events Among Individuals With CAC Score of 0 eTable 6. AUC C Statistics for Established Clinical Risk Tools and Self-rated Health for the Prediction of Hard CHD Events, Hard CVD Events, and All-Cause Death eTable 7. Comparison of AUC C Statistics for the Combination of CAC and EVGGFP vs PCE eTable 8. Comparison of AUC C Statistics for Combination of PCE and CAC With and Without Self-rated Health [file jamanetwopen-2-e188023-s001.pdf]

Supplementary Online Content

Orimoloye OA, Mirbolouk M, Uddin SMI, et al. Association between self-rated health, coronary artery calcium scores, and atherosclerotic cardiovascular disease risk: the Multi-Ethnic Study of Atherosclerosis (MESA). *JAMA Netw Open*. 2019;2(2):e188023. doi:10.1001/jamanetworkopen.2018.8023

eTable 1. Cumulative Incidence of Events by CAC Score Categories, Among Individuals Who Reported Excellent Self-Rated Health

eTable 2. Association Between CAC and Incident Events Among Individuals Who Reported Very Good Self-Rated Health

eTable 3. Association Between CAC and Incident Events Among Individuals Who Self-Reported Good Self-Rated Health

eTable 4. Association Between CAC and Incident Events Among Individuals Who Self-Reported Poor/Fair Health

eTable 5. Association Between Self-Rated Health and Incident Events Among Individuals With CAC Score of 0

eTable 6. AUC C Statistics for Established Clinical Risk Tools and Self-Rated Health for the Prediction of Hard CHD Events, Hard CVD Events, and All-Cause Death

eTable 7. Comparison of AUC C Statistics for the Combination of CAC and EVGGFP vs PCE

eTable 8. Comparison of AUC C Statistics for Combination of PCE and CAC With and Without Self-Rated Health

This supplementary material has been provided by the authors to give readers additional information about their work.

© 2019 Orimoloye OA et al. *JAMA Network Open*.

eTable 1. Cumulative Incidence of Events by CAC Score Categories, Among Individuals Who Reported Excellent Self-Rated Health

Excellent (N=1073)	CAC 0 (N=543)	CAC 1-100 (N=278)	CAC 100-400 (N=151)	CAC >400 (N=101)	*P* Value
**Hard CHD Events**	4, 0.74%	18, 6.5%	11, 7.3%	14, 13.9%	<.001
**Hard CVD Events**	6, 1.1%	32, 11.5%	17, 11.3%	17, 16.8%	<.001
**All-cause death**	9, 1.7%	15, 5.4%	10, 6.6%	13, 12.9%	<.001

© 2019 Orimoloye OA et al. *JAMA Network Open*.

eTable 2. Association Between CAC and Incident Events Among Individuals Who Reported Very Good Self-Rated Health

	All-cause death<br>HR (95% CI)	Hard CVD events<br>HR (95% CI)	Hard CHD events<br>HR (95% CI)
Age, gender and race/ethnicity adjusted			
CAC 0	**1.0**	**1.0**	**1.0**
CAC 1 - 99	1.2 (0.9 – 1.6)	1.5 (1.0 – 2.3)	2.1 (1.2 – 3.7)
CAC 100 - 399	1.5 (1.1 – 2.1)	3.7 (2.5 – 5.7)	6.4 (3.7 – 11.3)
CAC ≥ 400	1.7 (1.2 – 2.3)	2.7 (1.7 – 4.3)	5.1 (2.7 – 9.5)
❖ CAC (Yes/No)	1.4 (1.1 – 1.8)	2.2 (1.5 – 3.1)	3.4 (2.1 – 5.7)
❖ Ln (CAC + 1)	1.1 (1.1 – 1.2)	1.2 (1.1 – 1.3)	1.3 (1.2 – 1.4)
Risk factor adjusted [a]			
CAC 0	**1.0**	**1.0**	**1.0**
CAC 1 - 99	1.2 (0.9 – 1.6)	1.5 (1.0 – 2.2)	1.9 (1.0 – 3.5)
CAC 100 - 399	1.3 (0.9 – 1.9)	3.1 (2.0 – 4.9)	5.2 (2.9 – 9.4)
CAC ≥ 400	1.6 (1.2 – 2.3)	2.3 (1.4 – 3.8)	4.2 (2.1 – 8.1)
❖ CAC (Yes/No)	1.3 (1.0 – 1.7)	2.0 (1.4 – 2.9)	3.0 (1.7 – 5.0)
❖ Ln (CAC + 1)	1.1 (1.0 – 1.1)	1.2 (1.1 – 1.3)	1.3 (1.2 – 1.4)

a- Models adjusted for age, sex, race/ethnicity, hypertension, diabetes mellitus, use of lipid lowering medications, cigarette smoking, and family history of CVD.

© 2019 Orimoloye OA et al. *JAMA Network Open*.

eTable 3. Association Between CAC and Incident Events Among Individuals Who Self-Reported Good Self-Rated Health

	All-cause death<br>HR (95% CI)	Hard CVD events<br>HR (95% CI)	Hard CHD events<br>HR (95% CI)
Age, gender and race/ethnicity adjusted			
CAC 0	1 [Reference]	1 [Reference]	1 [Reference]
CAC 1 - 99	1.3 (1.0 – 1.6)	1.6 (1.2 – 2.3)	2.2 (1.4 – 3.4)
CAC 100 - 399	1.5 (1.2 – 2.0)	2.5 (1.8 – 4.0)	3.2 (2.0 – 5.2)
CAC ≥ 400	2.2 (1.7 – 2.9)	4.4 (3.0 – 6.5)	6.6 (4.1 – 10.6)
❖ CAC (Yes/No)	1.5 (1.2 – 1.9)	2.2 (1.6 – 2.9)	3.0 (2.0 – 4.4)
❖ Ln (CAC + 1)	1.1 (1.1 – 1.2)	1.2 (1.2 – 1.3)	1.3 (1.2 – 1.4)
Risk factor adjusted [a]			
CAC 0	1 [Reference]	1 [Reference]	1 [Reference]
CAC 1 - 99	1.2 (0.9 – 1.6)	1.4 (1.0 – 2.0)	2.0 (1.2 – 3.1)
CAC 100 - 399	1.3 (1.0 – 1.8)	2.2 (1.5 – 3.2)	2.9 (1.8 – 4.8)
CAC ≥ 400	2.0 (1.5 – 2.6)	3.5 (2.3 – 5.2)	5.3 (3.2 – 8.9)
❖ CAC (Yes/No)	1.4 (1.1 – 1.7)	1.9 (1.4 – 2.6)	2.6 (1.7 – 3.9)
❖ Ln (CAC + 1)	1.1 (1.0 – 1.1)	1.2 (1.1 – 1.3)	1.3 (1.2 – 1.3)

a- Models adjusted for age, sex, race/ethnicity, hypertension, diabetes mellitus, use of lipid lowering medications, cigarette smoking, and family history of CVD.

© 2019 Orimoloye OA et al. *JAMA Network Open*.

eTable 4. Association Between CAC and Incident Events Among Individuals Who Self-Reported Poor/Fair Health

	All-cause death<br>HR (95% CI)	Hard CVD events<br>HR (95% CI)	Hard CHD events<br>HR (95% CI)
Age, gender and race/ethnicity adjusted			
CAC 0	1 [Reference]	1 [Reference]	1 [Reference]
CAC 1 - 99	1.1 (0.8 – 1.7)	2.4 (1.3 – 4.6)	2.3 (1.0 – 5.4)
CAC 100 - 399	1.4 (0.9 – 2.2)	2.9 (1.4 – 5.9)	3.5 (1.4 – 8.8)
CAC ≥ 400	1.6 (1.0 – 2.5)	3.9 (1.9 – 7.9)	3.3 (1.3 – 8.6)
❖ CAC (Yes/No)	1.3 (0.9 – 1.8)	2.8 (1.6 – 5.0)	2.8 (1.3 – 5.9)
❖ Ln (CAC + 1)	1.1 (1.0 – 1.2)	1.2 (1.1 – 1.3)	1.2 (1.1 – 1.4)
Risk factor adjusted [a]			
CAC 0	1 [Reference]	1 [Reference]	1 [Reference]
CAC 1 - 99	1.0 (0.6 – 1.5)	2.1 (1.1 – 4.2)	1.9 (0.8 – 4.7)
CAC 100 - 399	1.1 (0.7 – 1.8)	2.0 (0.9 – 4.3)	2.3 (0.8 – 6.5)
CAC ≥ 400	1.3 (0.8 – 2.1)	3.1 (1.5 – 6.4)	2.6 (0.9– 7.4)
❖ CAC (Yes/No)	1.1 (0.8– 1.6)	2.3 (1.3 – 4.2)	2.1 (0.9 – 4.9)
❖ Ln (CAC + 1)	1.1 (1.0 – 1.1)	1.2 (1.1 – 1.3)	1.2 (1.0 – 1.3)

a- Models adjusted for age, sex, race/ethnicity, hypertension, diabetes mellitus, use of lipid lowering medications, cigarette smoking, and family history of CHD.

© 2019 Orimoloye OA et al. *JAMA Network Open*.

eTable 5. Association Between Self-Rated Health and Incident Events Among Individuals With CAC Score of 0

	All-Cause Mortality<br>HR (95% CI)	Hard CVD events<br>HR (95% CI)	Hard CHD events<br>HR (95% CI)
Age, Race and Gender adjusted			
Poor/Fair	1 [Reference]	1 [Reference]	1 [Reference]
Good	**0.61 (0.44 – 0.83)**	1.09 (0.65 – 1.82)	0.98 (0.48 – 1.97)
Very Good	**0.53 (0.37 – 0.75)**	0.85 (0.49 – 1.49)	0.63 (0.29 – 1.37)
Excellent	**0.26 (0.16 – 0.43)**	**0.20 (0.08 – 0.52)**	**0.21 (0.06 – 0.70)**
Risk factor adjusted*			
Poor/Fair	1 [Reference]	1 [Reference]	1 [Reference]
Good	**0.68 (0.48 – 0.94)**	1.28 (0.75 – 2.21)	1.19 (0.56 – 2.51)
Very Good	**0.58 (0.40 – 0.84)**	1.15 (0.64 – 2.07)	0.89 (0.39 – 2.04)
Excellent	**0.30 (0.18 – 0.51)**	**0.32 (0.12 – 0.85)**	0.37 (0.11 – 1.27)

*****Models adjusted for age, race/ethnicity, gender, hypertension, diabetes mellitus, use of lipid-lowering medications, cigarette smoking and family history of CHD.

© 2019 Orimoloye OA et al. *JAMA Network Open*.

eTable 6. AUC C Statistics for Established Clinical Risk Tools and Self-Rated Health for the Prediction of Hard CHD Events, Hard CVD Events, and All-Cause Death

	Hard CHD events	Hard CVD events	All-cause death
ASCVD risk score	**0.712**	**0.718**	**0.777**
CAC score	**0.725**	**0.693**	**0.685**
SRH	**0.542**	**0.546**	**0.573**

***SRH measured using the EVGGFP measure of overall health.**

© 2019 Orimoloye OA et al. *JAMA Network Open*.

eTable 7. Comparison of AUC C Statistics for the Combination of CAC and EVGGFP vs PCE

	PCE + CAC	PCE + CAC + SRH	p-value
Hard CHD	**0.751**	**0.753**	**0.39**
Hard CVD	**0.739**	**0.741**	**0.18**
All-cause death	**0.779**	**0.781**	**0.13**

***SRH measured using the EVGGFP measure of overall health.**

© 2019 Orimoloye OA et al. *JAMA Network Open*.

eTable 8. Comparison of AUC C Statistics for Combination of PCE and CAC With and Without Self-Rated Health

	SRH + CAC	PCE	p-value
Hard CHD events	**0.734**	**0.712**	**0.09**
Hard CVD events	**0.706**	**0.717**	**0.31**
All-cause death	**0.706**	**0.776**	**<0.001**

***SRH measured using the EVGGFP measure of overall health.**

© 2019 Orimoloye OA et al. *JAMA Network Open*.
